# Inerting Waste Al Alloy Dust with Natural High Polymers: Sustainability of Industrial Waste

**DOI:** 10.3390/ma15165540

**Published:** 2022-08-12

**Authors:** Bo Liu, Wenjing Yin, Kaili Xu, Yuyuan Zhang

**Affiliations:** 1 College of Resources and Civil Engineering, Northeastern University, Shenyang 110819, China; 2 College of Aviation Engineering, Weifang Engineering Vocational College, Qingzhou 262500, China

**Keywords:** dust explosion, Al alloy dust, natural high polymers, waste reuse, metal inerting

## Abstract

A large amount of waste dust will be produced in the process of metal grinding, resulting in a waste of resources and environmental pollution. Therefore, we present a new method of inerting waste aluminum (Al) alloy dust for recycling purposes. Three natural high polymers—starch, pectin, and hydroxypropyl cellulose—were selected to inert waste metal dust in order to prevent the alloy from hydrolyzing and keep the dust pure enough for reuse. The particles of the Al base alloy before and after dust reaction were characterized by scanning electron microscopy (SEM), X-ray diffraction (XRD), and Fourier transform infra-red (FTIR), and the relevant reaction mechanism was clarified. The hydrogen evolution test indicated that, across the temperature interval of 313–333 K, 0.75 wt% pectin inerted hydrogen evolution most efficiently (90.125%). XRD analysis indicated that the inerted product is composed of Al monomer and Al_3_Mg_2_, with no detectable content of Al hydroxide. The purity of the Al alloy dust was preserved. SEM and FTIR analyses indicated that the -OH, -COOH, and -COOCH_3_ functional groups in the high polymer participated in the coordination reaction by adsorbing on the surface of the waste Al alloy particles to produce a protective film, which conforms to Langmuir’s adsorption model. Verification of the inerted Al alloy dust in industrial production confirmed the possibility of reusing waste Al alloy dust. This study provides a simple and effective method for recycling waste Al alloy dust.

## 1. Introduction

Al is a very important nonferrous metal in the modern society, whose fine attributes have aroused increasing consumption and demand for Al products over the past decades. Restricted by the limited availability of Al resources, in addition to extensive mining of raw Al, researchers are increasingly interested in how to reuse waste Al materials [1]. In fact, it has long been a debated issue within the science and industry world whether it is desirable to treat the waste from Al recycling and how to realize this treatment. Unfortunately, there is yet no sophisticated recycling process in place. Many waste Al materials are land filled as industrial waste [2]. Cheol woo [3] confirmed that metal dust waste in the Korean manufacturing industry carries the risk of fire and explosion. According to statistics, the United Kingdom spends EUR 80 million dealing with waste Al materials. Japan disposes 350,000 tonnes of scum per year at the unit cost of USD 200–300 per tonne [4]. For many countries, this is not only an economic burden but also a waste of resources. Studies have demonstrated that the energy needed by the regenerated or secondary Al is just 5–20% of that needed by the produced Al [5]. The economy and the benefit of recycling Al are prominent.

Presently, research works are mostly concentrated around how to recycle bulk Al waste, having reported impressive results: fluidized bed, pulse atomization recovery, electrorefining, and many more [6,7]. Zhang et al. [8] proposed a new method of recycling waste magnesium alloy dust after treatment. Uan et al. [9] developed a new method of generating H_2_ gas in NaCl aqueous solution and achieved an H_2_ generation rate of 432.4 mL min^−1^. In Al material production and treatment, a lot of waste Al alloy dust is produced. These dust particles are basically a few micrometers to tens of micrometers in size. When exposed to air or water, they will be quickly oxidized into Al hydroxide, degrading the performance of Al and badly limiting the reuse of Al alloy [10]. These waste Al materials are mostly disposed as industrial waste rather than recycled as a reusable material. Environmentally, the existence of Al hydroxide will disrupt the acid-base balance of the soil and inhibit the growth of farm crops [11,12]. The UNEP “Basel Convention on the Control of the Border Transfer and Disposal of Hazardous Materials” also recommends rigorous control of hazardous wastes and secondary recycling of raw materials as a top priority for waste disposal [13]. If waste Al alloy dust were deactivated in a way that prevents the generation of oxides or hydroxides and keeps the metal dust pure, it would be possible to reuse it in future.

In view of the existing problems in the treatment methods of waste metal dust in the world at present, a method of inerting and collecting waste metal dust by adding an inerting agent into the wet dust collector is proposed. This method is advantageous because it is simple to operate, maximizes the purity of metal dust, prevents the generation of hydrogen, and reduces the risk of hydrogen explosion in the wet dust collector. In this study, we select three natural high polymers—starch (PS), pectin (PE), and hydroxypropyl cellulose (HC)—as inerting agents for waste metal dust [14,15,16]. The “Registration, Evaluation, Authorization and Restriction of Chemicals” (REACH) and the Paris Commission (PARCOM) rules on new chemicals are simple but clear: chemicals must be green [17]. Greenness is defined as non-biotic accumulation, biodegradation, and zero or minimal marine toxicity. Natural polymers are one of the few compounds meeting the REACH and PARCOM standards. As these polymers are environmentally compatible and highly available, they are very cost effective. Their intrinsic stability and multiple adsorption centers also qualify them as an inhibitor. These unique polymers have been extensively reported as a corrosion inhibitor due to their huge functional groups and surface complexation with metal ions. By covering the surface area of the metal in aqueous medium, these complexes effectively “cover” the surface and protect it against corrosive molecules or ions. In this study, we explore how three high polymers inert waste Al alloy dust. Our purpose is to develop a simple, potentially cost-effective technique, capable of preserving the performance of Al alloy dust while reducing its risk and provide a possibility of reusing waste Al alloy dust. Compared with previous studies, this paper provides a simple and potentially cost-effective conversion coating technology to protect the performance of Al alloy powder and reduce its risk. It provides a possibility for the processing, storage, transportation, and recycling of waste Al alloy dust.

## 2. Materials and Methods

### 2.1. Materials

**Figure 1:**
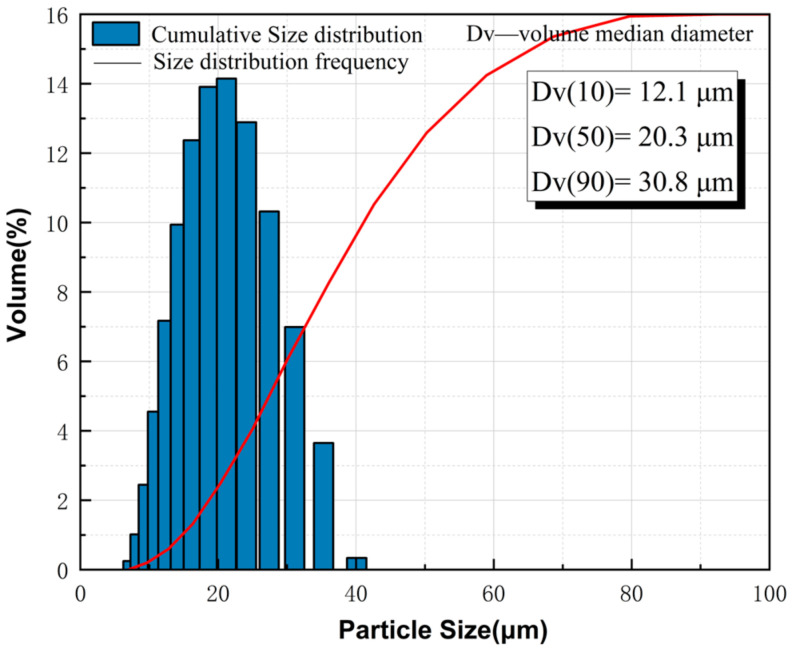
Particle size distribution of waste Al alloy dust.

The waste Al alloy dust (Al 89.37 wt%, Mg 10.586 wt%, Fe 0.0368 wt%, Si 0.0069 wt%, Cu 0.0013 wt%) used for the test came from the industrial waste collected in BMW’s grinding plant. The particle size distribution in the waste dust was characterized with a laser diffractometer (Mastersizer^®^2000), as shown in Figure 1. The median size of the waste Al alloy dust was around 20.3 μm. The potato starch, pectin (from apple), and hydroxypropyl cellulose were sourced from Macklin. All reagents were of analytical grade, so no further purification or differentiation was needed. Deionized water was used throughout the test.

### 2.2. Inerting Efficiency Measurement

**Figure 2:**
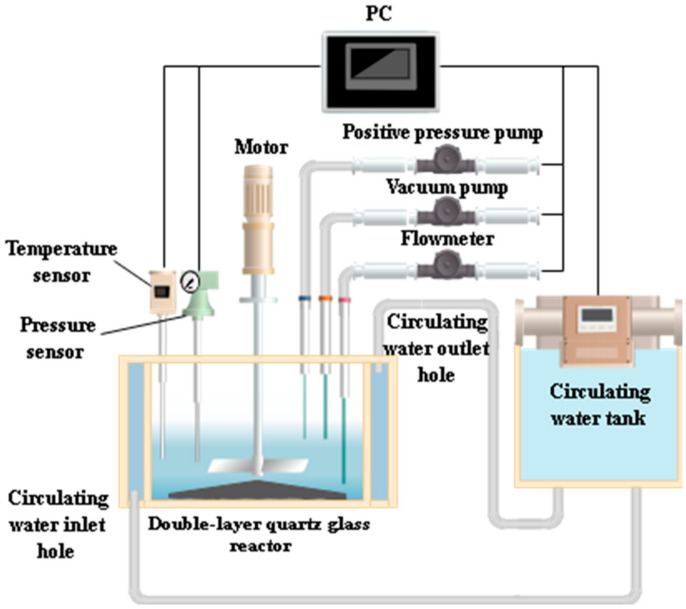
Automated hydrogen tester.

Al is an active metal that quickly hydrolyzes into hydrogen when placed in an aqueous solution. Hence, by measuring the hydrogen conversion parameter of waste Al alloy dust, we can know to what degree the metal dust is inerted and the Al alloy is oxidized. The integrated test was conducted in a proprietary automated hydrogen tester, as shown in Figure 2. The design and principle of this tester are described in our previous studies [18,19]. The formula for calculating the hydrogen conversion parameter α is given by Equation (2), which builds on the isobaric gas equation (Equation (1)). If the reactor volume V is fixed, R is a constant, and T is invariable in the reaction, the pressure P will be directly proportional to the amount of the reaction-generated hydrogen material, which then describes the progress of the reaction. This gives the hydrogen conversion parameter α of Al alloy dust particles in different solutions at any time.

(1) PV=nRT

(2) $$\alpha =\frac{\left(P_{0}-P_{\mathrm{initial}}\right)\left(V_{0}-V_{\mathrm{solution}}\right)}{n_{0}\mathrm{RT}}$$

where P—pressure, Pa; V—gas volume, m^3^; T—temperature, k; n—the amount of substance in the gas, mol; R—the molar gas constant, J/mol·k.

α—hydrogen evolution; P_0_—gas pressure in the reactor, kPa; Pinitial—initial pressure value of the reactor, kPa; V_0_—Reactor volume, L; V_solution_—the volume of the solution added into the reactor, L; n_0_—the mole of Al powder added into the reactor, mol.

First, 200 mL PS, PE, and HC of different concentrations was each reacted with 1.5 g waste Al alloy dust for 12 h at the initial atmospheric pressure of 100 kPa and initial temperature of 323 K to test hydrogen production. Next, the film-forming stability of the high polymers on the surface of Al alloy dust particles was tested for different temperatures. These two steps are necessary, since they evaluate the inerting effect and consider the possibility of a reduced inerting effect in a complex production environment. In order to ensure accuracy and preclude exposure to other factors, each hydrogen evolution test was repeated three times. Each group of the test was preceded by a 12 h air tightness test under the initial pressure of 300 kPa and the temperature of 323 K. If the pressure change was within ±0.1 kPa in 12 h, the instrument was deemed airtight enough to produce an eligible test.

### 2.3. Characterization Test

Scanning electron microscopy (SEM) and energy-dispersive spectrometry (EDS) were used to characterize the topographical change of the Al alloy dust particles before and after reaction, as well as the atomic percentage of Al and O in the product. The current of the focusing beam of EDS was 10uA, and the voltage was 15 kV. In order to better understand the phase of the product, X-ray diffractometry (XRD) was used to analyze the products of Al alloy dust with different inerting agent solutions. The initial voltage and current settings were 40 kV and 40 mA. During the experiment, 2θ was set to range from 5° to 90°. The scanning step was 0.01°. The scanning speed was 0.02°s^−1^. Fourier transform infra-red (FTIR) was used to analyze the functional groups in the product inerted by the inerting agent.

### 2.4. Industrial Verification

Industrial verification is a necessary step to determine whether it is possible to reuse the inerted waste Al alloy dust. According to the hydrogen evolution test result, the Al alloy dust was kept in 0.75 wt% PE solution for 12 h, then washed with deionized water, and dried in a vacuum dryer for 12 h. The prepared metal dust was then tested for further use in Minxin Powder Metallurgy Company (Guangdong, China), our partner manufacturer, which is a producer and OEM manufacturer of auto fittings for many auto brands. We tried to reproduce the inerted Al alloy dust into auto seat fittings and tested the mechanical property of the produced fittings to industry standard. We tested the strength of the improved Al alloy block in the collaborative industrial laboratory, measuring its Vicker hardness, room-temperature quasistatic tensile strength, and compressive mechanical behavior.

## 3. Results and Discussion

### 3.1. Inerting Efficiency

#### 3.1.1. Effect of Inerting Agent Concentration

**Figure 3:**
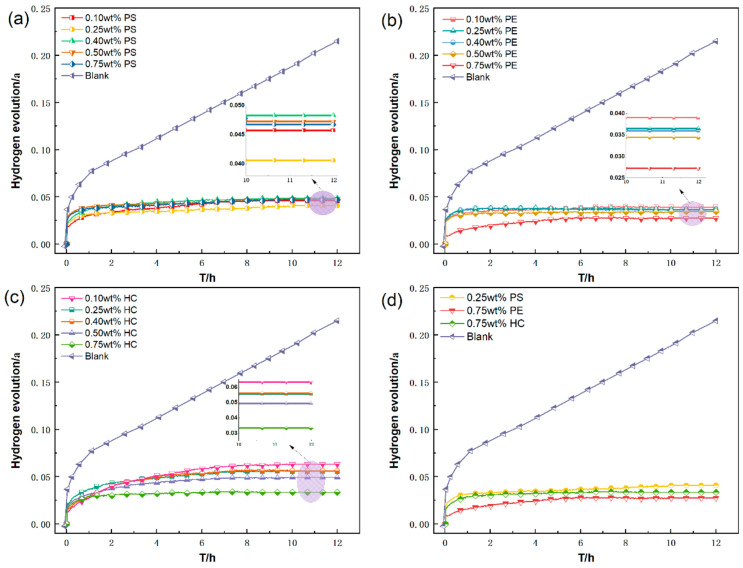
Hydrogen evolution curves and comparison curve (**d**) of Al alloy dust in (**a**) PS, (**b**) PE, (**c**) HC inerting agent solutions.

Figure 3 compares the hydrogen evolution curves of waste Al alloy dust in different inerting agent solutions at different concentrations. We also used a blank group without an inerting agent. From these graphs, before an inerting agent is added, the hydrogen evolution curve of waste Al alloy dust rises straight up and is still rising 12 h later. As has been demonstrated by our previous study [20], the hydrogen evolution of Al alloy dust is a continuous, slow process that can last for 72 h. Compared with the control group, PS, PE, and HC greatly inhibit the hydrogen evolution of Al alloy dust. The optimal inhibition concentration of PS is 0.25 wt%, at which α is as low as 0.04058. However, beyond 0.25 wt%, the hydrogen inhibition begins to reduce with increasing PS concentration. This is because, as the concentration increases, the solubility and surface binding strength of molecular starch are reduced, causing the corrosion inhibition efficiency to reduce too [21]. The inerting efficiency of PE increases with increasing concentration. The optimal inhibition concentration is 0.75 wt%, at which α is as low as 0.02123. From Figure 3b, although increased PE concentration improves the inerting efficiency, the hydrogen evolution curves under different concentrations are close to each other. The α values are not much different. Obviously, increased concentration does not improve the inerting efficiency for Al alloy dust significantly. The inerting efficiency of HC for waste Al alloy dust also increases greatly with increasing concentration. The optimal inhibition concentration is 0.75 wt%, at which α is as low as 0.03442. The concentration of HC affects the inerting efficiency for waste Al alloy considerably. At low concentrations, its inerting efficiency is not as good as PS and PE.

Figure 3d compares the hydrogen evolution of Al alloy dust with PS, PE, and HC at the optimal concentration. We can see that 0.75 wt% PE performs better than 0.25 wt% PS and 0.75 wt% HC, resulting in the lowest hydrogen evolution. Therefore, by inerting efficiency, PE > HC > PS. The hydrogen inhibition efficiency (ω) of these inerting agents for Al alloy dust was calculated by Equation (3). The result indicated that PE has the highest inhibition efficiency, 90.125%, compared to 81.125% for PS and 83.991% for HC. In terms of hydrogen inhibition, PE performs the best.

(3) $$\omega =\frac{\alpha_{\mathrm{blank}}-\alpha_{\mathrm{inh}}}{\alpha_{\mathrm{blank}}}\times 100\%$$

where $\alpha_{\mathrm{blank}}$ and $\alpha_{\mathrm{inh}}$ represent the hydrogen conversion rate without and with an inhibitor, respectively.

#### 3.1.2. Effect of Temperature

**Figure 4:**
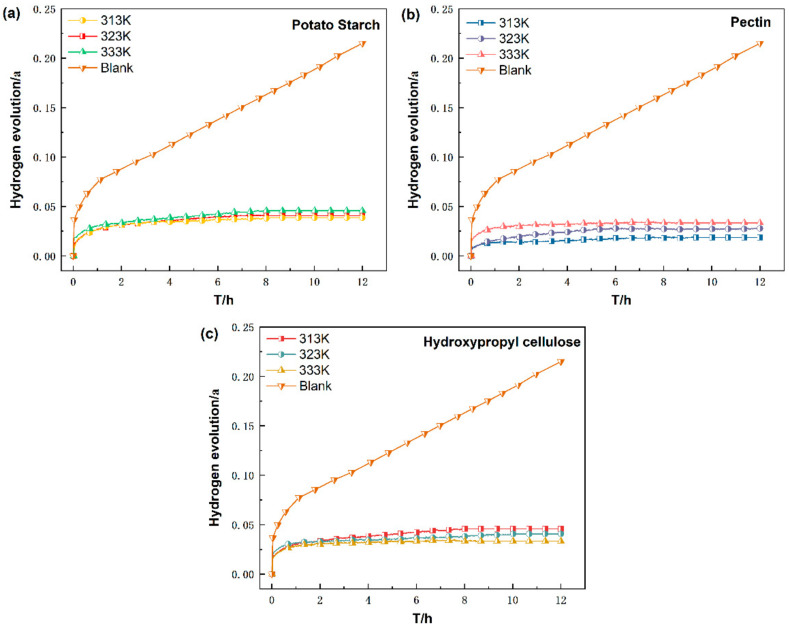
Hydrogen evolution curves of Al alloy dust in (**a**) PS, (**b**) PE, (**c**) HC inerting agent solutions under different temperatures.

Figure 4 compares the hydrogen evolution curves of Al alloy dust in the three inerting agent solutions at their optimal concentration across the temperature interval of 313–333 K. The inerting efficiency of PS and PE reduces slightly with increasing temperature. Increased temperature limits the adsorption of the inerting agent on the Al particle surface. For PS, the inerting efficiency is less affected by temperature. For PE, the inerting efficiency (89.054%) remains high even under 333 K. For HC, the inerting efficiency increases with increasing temperature, reaching 85.396% under 333 K compared to 81.125% under 323 K. The inerting agent HC can retard corrosion through chemical adsorption under high temperature. This is attributable to the presence of carboxyl groups in HC, which, in the spectral chemistry series, are thought of as a better ligand than hydroxyl [22].

#### 3.1.3. Chemical Kinetics Model

Previous research works have proved that the fluid–solid reaction can be explained by a shrinking core model considering three stages: external diffusion, internal diffusion, and chemical reaction [23,24]. The reaction of Al alloy dust particles in aqueous solutions is not subject to external diffusion. As there is no diffusion of the reacted material in the liquid film around the solid particles, the reaction between the Al alloy particles and water is subject only to internal diffusion and chemical reaction. Furthermore, as the inerting agents primarily work on the surface of Al alloy dust particles without involving internal diffusion, we only considered chemical reaction. Table 1 gives the k values of the Al alloy in different inerting agent solutions, calculated by Equation (4).

The calculation result of the shrinking core model is the same as the data obtained by the hydrogen evolution test. In the PS (0.25 wt%) solution, the minimum reaction rate (k) of Al alloy dust is 8.8889 × 10^−4^. When PE is used, k can be 4.6800 × 10^−5^. The reaction rate is close to 0 and is lower than when the other two inerting agents are used. Obviously, PS can best protect Al alloy dust against oxidation in the solution, further verifying that PE inerts Al alloy dust more effectively than PS and HC.

#### 3.1.4. Adsorption Isotherm

In order to understand how inhibition works, the surface coverage of the inerting agents at different concentrations was analyzed across the temperature interval of 323–333 K. Many authors have demonstrated that adsorption isotherm can describe the interaction between the inhibitor molecules and the alloy surface [23], defined as the variation of the adsorption strength of the inhibitor on the alloy surface as a function of inhibitor concentration. In an aqueous solution, the surface of Al alloy particles is covered by water molecules; in an inhibitor solution, the inhibitor molecules adsorb on the surface of the alloy particles in place of water molecules. Surface coverage was tested by fitting different adsorption isotherms, such as Langmuir, Temkin, Frumkin, and Freundlich. The results indicated that the Langmuir curve (Equation (4)) coincided with the test value obtained by the hydrogen evolution test. For HC, the correlation of the Freundlich curve was 0.992, suggesting that the adsorption involved both physical and chemical processes. Similar observation was made on many organic inhibitors, including HC. One possible explanation for the 1.0 slope deviation is the interaction among the inhibitor molecules adsorbed on the metal surface [25,26].

(4) $$\frac{C}{\theta }=\frac{1}{K}+C$$

where C—mass concentration of inhibitors, g L^−1^, K—adsorption equilibrium constant, L g^−1^, and θ—surface coverage.

### 3.2. SEM and EDS

**Figure 5:**
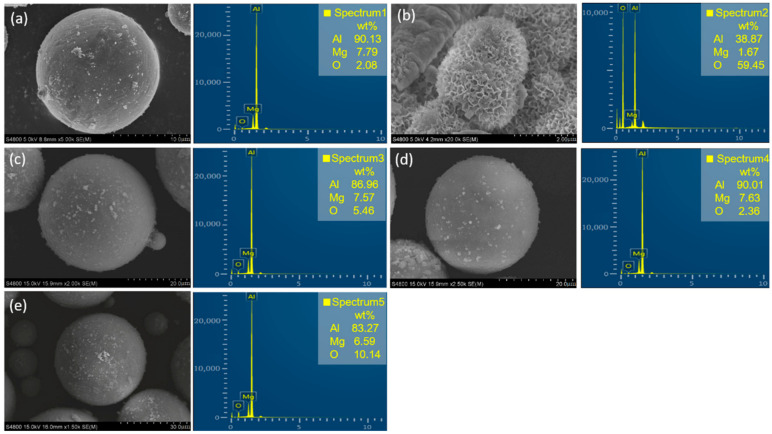
SEM micrographs and EDS of Al alloy dust before (**a**) and after (**b**) reaction in blank solution and those containing various inhibitor concentrations: 0.25 wt% PS (**c**), 0.75 wt% PE (**d**), 0.75 wt% HC (**e**).

Figure 5 compares the topographies of Al alloy dust before and after reaction in different inerting agent solutions. In Figure 5a, before reaction, the metal particle surface is smooth and ellipsoid. EDS indicates high Al and Mg contents (90.13%, 7.79%). After reaction in the aqueous solution, the particle surface becomes flocculent as a result of the outward diffusion of hydrogen generated by Al–water reaction. By then, Al and Mg are extensively consumed. The O content increases to 59.45%. The particle surface is wrapped by Al (OH)_3_ and Mg (OH)_2_. Therefore, without adding an inerting agent, as the waste metal particles contain many hydroxides, they cannot but be disposed as waste. This would mean a hazard to the environment and a waste of resources. When an inerting agent is added, the metal particle surface is covered by the protective film produced by the inerting agent. This blocks the Al inside from water molecules and makes the particle surface smooth, further preserving the property of the metal particles. The Al and Mg contents are basically unchanged. From the SEM result, all three inerting agents can keep the particle surface smooth. From the EDS result, the metal dust reacted in the HC solution has a high O content (10.14%). Although HC can inhibit most Al from participating in the hydrogen evolution reaction, the protective film produced cannot cover the entire particle surface. A small part of the Al matrix is still corroded.

Among the three inerting agents, Al alloy dust particles behave the best in PE. The particle surface is smooth, without flocculent precipitation. The Al and Mg contents can be as high as 90.01%, 7.63%, which is not much different from before the reaction. The protective film produced by PE can cover the entire metal particle surface. This blocks the Al particles from water and prevents them from hydrolyzing. As the metal particles inerted by PE still maintain their metallic property, it is possible to reuse them in further production.

### 3.3. Inerting Product

**Figure 6:**
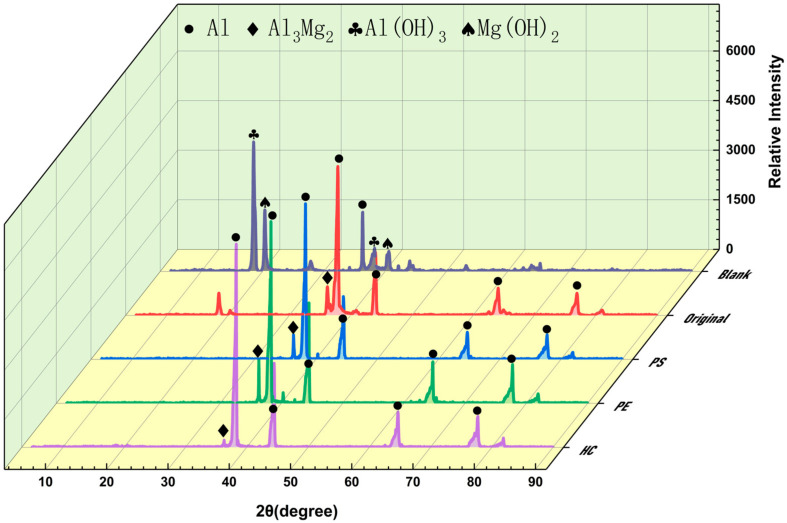
XRD curves of Al alloy before and after dust reaction and after inerting agent.

In order to prove the observations obtained by SEM and EDS analyses and verify the main components in the inerted metal dust particles, we tested the Al alloy dust particles before and after reaction by XRD, as shown in Figure 6. From the original curves, before the reaction, the diffraction peaks are primarily related to the Al monomer and Al_3_Mg_2_. According to previous research works [27], for the Al–*x*Mg alloys, when x > 56%, the main alloy components are Mg and γ-alloy (Al_12_Mg_17_); when 35% < x < 56%, the main alloy components are β-alloy (Al_3_Mg_2_) and γ-alloy (Al_12_Mg_17_); when x < 35%, the main alloy components are Al and β-alloy (Al_3_Mg_2_ for β-alloy). Therefore, the alloy in the Al alloy dust is a β-alloy. β-alloy is hydrogen absorptive, as has been demonstrated by our previous study [28]. Moreover, no diffraction of an Al oxide or hydroxide was detected. After it is produced, waste dust will be drawn into the wet dust collector, during which it is exposed to air for too short a time to become oxidized. By then, the metal dust is quite pure. After it comes into the wet dust collector (blank), many Al (OH)_3_ and Mg (OH)_2_ diffraction peaks appear in the product. The diffraction peak of the Al monomer also shrinks greatly. Chemical reactions described by Equations (5)–(7) occur in Al alloy dust particles in water. A lot of Al and Al_3_Mg_2_ participate in the reactions, reducing the water molecules to H^+^. One part of the H^+^ gathers on the metal particular surface to form H_2_ and separates out. Another part adsorbs on the surface of Mg^+^ to form MgH^+^. Eventually, Al (OH)_3_ and Mg (OH)_2_ are generated to wrap on the metal particular surface.

(5) $$2\mathrm{Al}+6H_{2}O\to 2\mathrm{Al}{\left(\mathrm{OH}\right)}_{3}+3H_{2}↑$$

(6) $${\mathrm{Al}}_{3}{\mathrm{Mg}}_{2}+2H_{2}\to 2{\mathrm{MgH}}_{2}+3\mathrm{Al}$$

(7) $${\mathrm{MgH}}_{2}+2H_{2}O\to \mathrm{Mg}{\left(\mathrm{OH}\right)}_{2}+2H_{2}↑$$

From the diagram, when the inerting agents PS, PE, and HC are added, the diffraction peaks of the reaction product show roughly the same trend as those of the Al alloy dust before reaction—in both cases, the diffraction peaks are only related to the Al monomer and Al_3_Mg_2_. Obviously, all three inerting agents can virtually preserve the purity of Al alloy dust particles. By area of diffraction peak, the Al monomer in the HC product is smaller than in the other two agents; the diffraction peak curve of PS is the most coincidental with that of the original metal dust particles. Furthermore, although the EDS result indicated that after HC reaction, the surface of Al alloy dust particles contains some O, it was not detected in XRD. The oxygen content produced is too limited to detect an obvious diffraction peak. The XRD result confirms that after adding an inerting agent, Al alloy dust particles are composed of the Al monomer and β-alloy with no other impurities. The purity of the metal dust is preserved.

**Figure 7:**
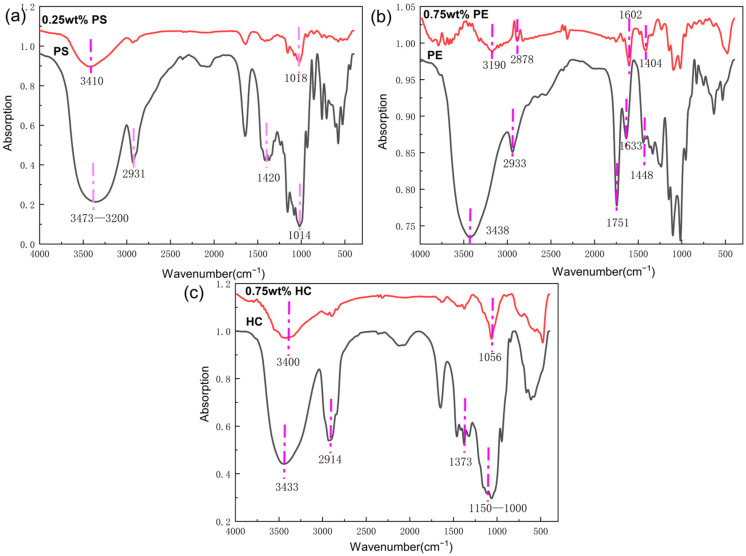
FTIR curves of inerting agent (**a**) PS, (**b**) PE, and (**c**) HC and its reaction products.

**Figure 8:**
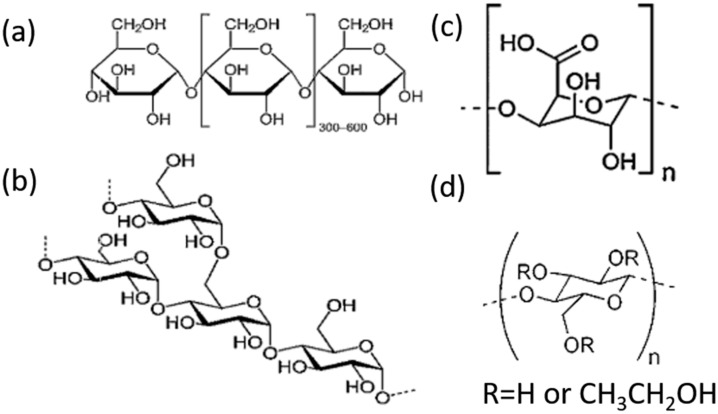
Chemical structures of the amylose (**a**), amylopectin (**b**), Pectin (**c**), and hydroxypropyl cellulose (**d**).

In order to explore the inerting mechanism, FTIR analysis was carried out on the three inerting agents and their reaction products, as shown in Figure 7. PS, as a polysaccharide produced by polymerizing glucose molecules, is a macromolecular carbohydrate. Data indicate that PS contains both amylose and amylopectin, with amylopectin taking a higher proportion. The former is a branchless helical structure comprising glucose monomer units connected together to form α-1,4 chains, which are connected to the epoxy atoms on the same side; the latter consists of 24–30 glucose residues connected end to end by α-1,4-glycosidic bonds and by α-1,6-glycosidic bonds at the branch, as shown in Figure 8a,b. Figure 7a shows the FTIR curves before and after PS is added. As can be seen, the broad absorption band at 3473–3200 cm^−1^ consists of multiple O-H stretching vibrations overlapped together; the characteristic peaks at 2931 cm^−1^ and 1420 cm^−1^ are attributed to the stretching vibration of CH_2_; the characteristic peak at 1014 cm^−1^ is a C-O-C stretching vibration; that at 1000–850 cm^−1^ is a C-O-C symmetric stretching vibration. When 0.25 wt% PS is used, the characteristic peak at 3473–3200 cm^−1^ narrows and shifts to the left, suggesting that part of the -OH participated in the coordination reaction and reduced the bond energy of O-H. The characteristic peaks at 2931 cm^−1^ and 1420 cm^−1^ disappear; the C-O-C characteristic peak at 1014–850 cm^−1^ weakens and shifts aside, suggesting that adsorption took place between C-O-C in PS and Al^3+^, as has been observed by other authors [29].

PE is an acid heteropolysaccharide composed of D-Galacturonic Acids (D-Gal-A) connected by α-1,4-glycosidic bonds. It consists mainly of -OH, C-H, and C-O-C functional groups, as shown in Figure 8c. In Figure 7b, the characteristic peak at 3483 cm^−1^ is a typical O-H stretching vibration; that at 2933 cm^−1^ is attributed to the C-H stretching vibration of the CH_2_ group; the spectral band at 1751 cm^−1^ can be attributed to an O-C-O stretching vibration. The PE sample also displays two vibration peak bands at 1380 cm^−1^ and 1300–1000 cm^−1^, which are, respectively, caused by the bending of C-H bonds and the stretching of C-O bonds. When 0.75 wt% PE is used, the characteristic peak at 3483 cm^−1^ narrows and shifts leftward to 3190 cm^−1^, suggesting that a lot of the OH in PE participated in the adsorption reaction. The characteristic peak at 1751 cm^−1^ disappears; those at 1633 cm^−1^ and 1448 cm^−1^ narrow and shift to the right. FTIR analysis indicated that OH, COOH, and COOCH_3_ are the main functional groups in the product that participated in the adsorption reaction. These groups contain one oxygen atom featuring lone pair electrons, which, as the adsorption center, inhibits the hydrogen evolution of Al alloy dust. Similar results have also been reported for PE by other authors [30,31].

HC and PS are both macromolecular polysaccharides composed of glucose, as shown in Figure 7c. The characteristic peak at 3433 cm^−1^ is an O-H stretching vibration; those at 2914 cm^−1^ and 1373 cm^−1^ are a variable angle vibration of CH_2_; the multiple peak bands between 1150 and 1000 cm^−1^ are the C-O-C and C-OH stretching vibration of saccharides. As the adsorption peaks of both groups are within the same interval, they are hardly distinguishable. When 0.75 wt% HC is used, the characteristic peak at 3433 cm^−1^ narrows and shifts to the lower band; the C-H stretching peaks at 2914 cm^−1^ and 1373 cm^−1^ almost disappear completely; the C-O characteristic peak at 1150–1000 cm^−1^ narrows and shifts to 1056 cm^−1^. The disappearance and shift of peaks indicate that many C-H bonds and ether functional group C-O bonds in HC participated in the coordination reaction, as has been noted in the literature [32].

### 3.4. Inerting Mechanism

**Figure 9:**
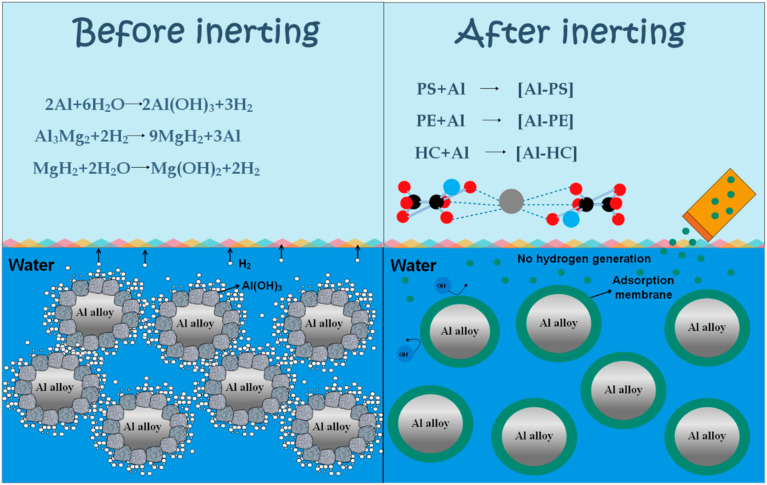
Inhibition mechanism of PS, PE, and HC.

Based on the test results, the inerting mechanisms of the three inerting agents for Al alloy dust were analyzed. From the FTIR result, natural polymers (PS, PE, HC) contain abundant hydroxyl and carboxyl functional groups. Each of these groups contains one oxygen atom featuring lone pair electrons, which, as the adsorption center, chelates with the surface of Al alloy dust particles. However, due to the long molecular chain of PS, it is easy to entangle, so the inhibition effect of PS is poor with the increase in content. As shown in the SEM micrographs (Figure 5), these high polymers can adsorb on the surface of Al alloy particles to produce a tight protective film that blocks the OH^−^ in water from Al^3+^ ions, as has been noted by previous research works [14,33]. The reaction mechanism is illustrated in Figure 9. The XRD result confirms that the inerting agents can prevent the generation of oxides and hydroxides. They are well able to preserve the purity of Al alloy dust and make reuse possible. As indicated by the hydrogen evolution test, PE is the best inhibitor for Al alloy dust. The low solubility and surface binding strength of molecular starches determine that they are not suitable as a corrosion inhibitor for extensive application. Neither is HC widely applicable, since it relies on high temperature to improve the inerting efficiency. Only PE can serve as an inexpensive green inerting agent for waste Al alloy dust.

### 3.5. Industrial Verification

In order to investigate the feasibility of reusing inerted Al alloy dust in industrial production, Al alloy dust particles reacted by 0.75 wt% PE were used for powder metallurgy processing at our partner manufacturer. The resulting Al alloy fittings were tested for mechanical property in an industrial laboratory and then compared with standard Al alloy fittings, as shown in Table 2. The mechanical property of the inerted Al alloy fittings was almost the same as the standard ones. The error was within the acceptable limit. The 90.5 HB hardness and 175.62 MPa tensile strength substantially conform to the requirements of the applicable standard. The tolerance measurement of the inerted Al alloy fittings showed: Al K: 90.13, Mg K: 7.58, C K: 0.15. O K: 2.14. No other detrimental effect was detected. The conformance of the inerted Al alloy fittings with the product standard for auto fittings demonstrates the feasibility of reusing waste Al alloy dust in further production.

## 4. Conclusions

We selected three natural high polymers (PS, PE, HC) as inerting agents for waste Al alloy dust for industrial reuse. Under 323 K, the inerting efficiency followed the PE > PS > HC sequence, at the optimal concentration of 0.25 wt%, 0.75 wt%, 0.75 wt%, respectively. The inerting agents performed well under 273 K. However, as the temperature increased, the inerting efficiency of HC improved a little due to chemical adsorption. SEM analysis indicated that the inerting agent formed a smooth protective film over the surface of waste Al alloy particles. FTIR analysis indicated that the protective film resulted from the adsorption between the lone pair electrons contained in the O of the OH, COOH, and COOCH_3_ in the high polymer, and the empty 3d orbits of Al atoms. The calculations showed that this conforms to the Langmuir adsorption isotherm, suggesting a physical rather than a chemical adsorption. Industrial verification showed that the Al alloy fittings fabricated from Al alloy dust inerted by 0.75 wt% PE are almost the same as standard Al alloy fittings in terms of mechanical property and purity. The inerting process described here is easy and pollution free. It can turn waste Al alloy into a useful byproduct rather than a potential environmental hazard. This reduces the financial cost to manufacturers and protect the environment. As the inerting process precludes the generation of hydrogen, it also limits the risk of hydrogen explosion. However, our study is only a preliminary verification of feasibility. To enable full industrial production, further detailed study has to be carried out as to the potential problems in the use of the inerted Al alloy fittings.

## Figures and Tables

**Table 1:** Reaction rate constants.

Corrosion Inhibitor	Concentration (wt%)	k (h^−1^)
PS	0.1 wt%	8.9009 × 10^−4^
	0.25 wt%	8.8889 × 10^−4^
	0.4 wt%	0.00159
	0.5 wt%	0.00156
	0.75 wt%	9.4697 × 10^−4^
PE	0.1 wt%	5.4730 × 10^−4^
	0.25 wt%	3.2068 × 10^−4^
	0.4 wt%	2.4066 × 10^−4^
	0.5 wt%	8.6672 × 10^−5^
	0.75 wt%	4.6800 × 10^−5^
HC	0.1 wt%	0.00181
	0.25 wt%	0.00144
	0.4 wt%	0.00106
	0.5 wt%	9.7387 × 10^−4^
	0.75 wt%	3.6469 × 10^−4^

**Table 2:** Comparison of mechanical properties between standard Al alloy fittings and inerted Al alloy fittings.

	Hardness	Tensile Strength	Maximum Compressive Strength	Elastic Modulus
Standard Al alloy fittings	91.6 HB	175.94 MPa	172.38 KN	39.68%
Inerted Al alloy fittings	90.5 HB	175.62 MPa	170.54 KN	38.99%

## Data Availability

Data sharing not applicable. No new data were created or analyzed in this study.
